# Intensive Care Unit-Acquired Weakness and Positioning-Related Peripheral Nerve Injuries in COVID-19: A Case Series of Three Patients and the Latest Literature Review

**DOI:** 10.3390/brainsci11091177

**Published:** 2021-09-06

**Authors:** Keiichi Hokkoku, Carmen Erra, Cristina Cuccagna, Daniele Coraci, Dario Mattia Gatto, Davide Glorioso, Luca Padua

**Affiliations:** 1 UOC Neuroriabilitazione ad Alta Intensità, Fondazione Policlinico Universitario A. Gemelli IRCCS, 00168 Rome, Italy; k1-hokkoku@hotmail.co.jp (K.H.); carmen.erra@hotmail.it (C.E.); danielecoraci@aol.com (D.C.); dario.gatto90@gmail.com (D.M.G.); davideglorioso@libero.it (D.G.); luca.padua@unicatt.it (L.P.); 2 Department of Neurology, Teikyo University School of Medicine, Tokyo 173-8605, Japan; 3 Department of Neurosciences, Università Cattolica del Sacro Cuore, 00168 Rome, Italy; 4 Department of Orthopaedics and Geriatrics, Università Cattolica del Sacro Cuore, 00168 Rome, Italy

**Keywords:** COVID-19, ICUAW, critical illness polyneuropathy, critical illness myopathy, peripheral nerve injury, personalized medicine

## Abstract

A subgroup of COVID-19 patients requires intensive respiratory care. The prolonged immobilization and aggressive treatments predispose these patients to develop intensive care unit-acquired weakness (ICUAW). Furthermore, this condition could increase the chance of positioning-related peripheral nerve injuries. On the basis of the latest literature review, we describe a case series of three patients with COVID-19 who developed ICUAW complicated by positioning-related peripheral nerve injuries Every patient presented sensorimotor axonal polyneuropathy and concomitant myopathy in electrophysiological studies. Furthermore, muscle MRI helped the diagnosis of ICUAW, showing massive damage predominantly in the proximal muscles. Notably, nerve ultrasound detected positioning-related peripheral nerve injuries, even though the concomitant ICUAW substantially masked their clinical features. During the acute phase of severe COVID-19 infection, most medical attention tends to be assigned to critical care management, and neuromuscular complications such as ICUAW and positioning-related peripheral nerve injuries could be underestimated. Hence, when starting post-ICU care for COVID-19 cases, the combination of electrophysiological and imaging studies will aid appropriate evaluation on the patients with COVID-19-related ICUAW.

## 1. Introduction

Intensive care unit-acquired weakness (ICUAW) is an acquired neuromuscular disorder in critical patients characterized by generalized muscle weakness and failure to wean from the ventilator. It consists of three subtypes: critical illness polyneuropathy (CIP); critical illness myopathy (CIM); and critical illness neuromyopathy (CINM), a combined form of CIP and CIM [1,2]. In general, ICUAW is seen in approximately 25–45% of critically ill patients and is associated with prolonged mechanical ventilation and hospitalization, increased mortality, and poor functional ability in post-ICU patients [1,2,3,4].

Since the coronavirus disease-19 (COVID-19) outbreak started, ICU departments have been under high pressure because approximately 15% of COVID-19 cases develop acute respiratory distress syndrome (ARDS) requiring ICU admission [5]. In this regard, several researchers have insisted that severe COVID-19 cases can be more predisposed to develop ICUAW than general ICU patients [5,6,7,8,9]. Firstly, COVID-19-related ARDS requires rigorous mechanical ventilation with deep sedation to avoid lung injury due to ventilator dyssynchrony, typically seen in this complication, and the condition could result in excessive immobilization, a major risk factor of ICUAW [5,6,8,10]. Furthermore, the characteristics of severe COVID-19 patients, such as older age, multiple comorbidities including diabetes, cytokine overproduction, and the use of corticosteroids for the treatment, could also contribute to developing ICUAW [5,6,7,8,11,12,13,14]. In light of these clinical aspects and the current pandemic situation, many researchers are concerned about the growing incidence of COVID-19-related ICUAW [6,7,13,14].

In addition to ICUAW, peripheral nerve injuries during ICU care have been reported as another possible neuromuscular complication in COVID-19 cases [15,16,17,18,19]. While peripheral nerve injury is a known complication in general ICU care, COVID-19 patients may be more prone to this complication due to intensive respiratory management, including prolonged immobilization and prone positioning, leading to unexpected compression and stretching of the nerves [15,16,17,20]. 

In this case series, we describe three COVID-19 patients with ICUAW complicated by peripheral nerve injury. We discuss the utility of electrophysiological studies, nerve ultrasound (NUS), and muscle MRI to evaluate the characteristic neuromuscular complication of COVID-19 on the basis of the latest literature review.

## 2. Case Presentation

### 2.1. Case 1

A 52-year-old man developed ARDS due to a COVID-19 infection requiring ICU admission. Because of the severe respiratory failure, he underwent long-term mechanical ventilation (60 days), intermittent prone positioning, and extracorporeal membrane oxygenation (ECMO). During the clinical course, he also developed septic shock and multiple organ failure. The first neurological evaluation performed on day 46 revealed generalized muscle weakness and atrophy, as well as absent tendon reflexes. Nerve conduction study (NCS) revealed sensorimotor polyneuropathy with a decreased compound muscle action potential (CMAP) in the right median nerve (0.7 mv) and decreased sensory nerve action potential (SNAP) in the right radial nerve (4.0 µv). Electromyography (EMG) revealed denervation potential in the right biceps brachii; the voluntary activity was not assessed due to the effect of sedation. These findings suggested the existence of ICUAW.

**Figure 1:**
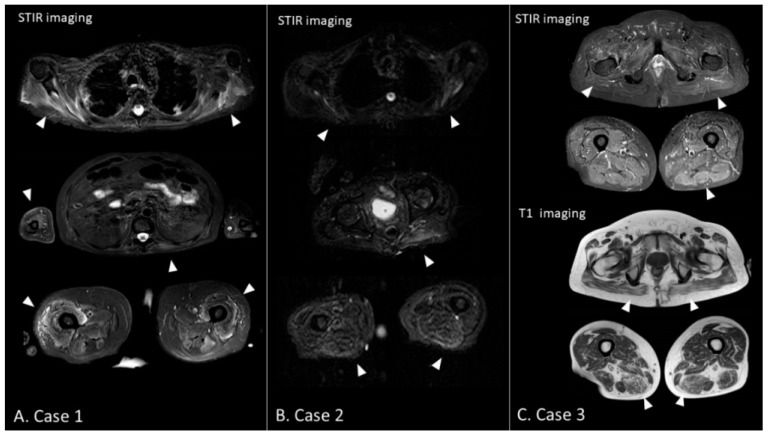
Muscle MRI findings. (**A**) Case 1: Hyperintense lesions are observed in the shoulder girdle muscles, biceps brachii, left paraspinal muscles, and anterior compartment of the thighs in STIR imaging (triangles). (**B**) Case 2: Hyperintense lesions are observed in the shoulder girdle muscles, left gluteus maximus, and posterior compartment of the thighs in STIR imaging (triangles). Furthermore, marked atrophy is seen in the thighs (triangles). (**C**) Case 3: In STIR imaging, hyperintense lesions are observed in the limb-girdle muscles, including the gluteus maximus and posterior compartment of the left thigh. In T1 imaging, hyperintense lesions are more prominent than in the STIR imaging, suggesting fatty infiltration, a finding of chronic phase ICUAW (triangles). STIR, short T1 inversion recovery.

**Figure 2:**
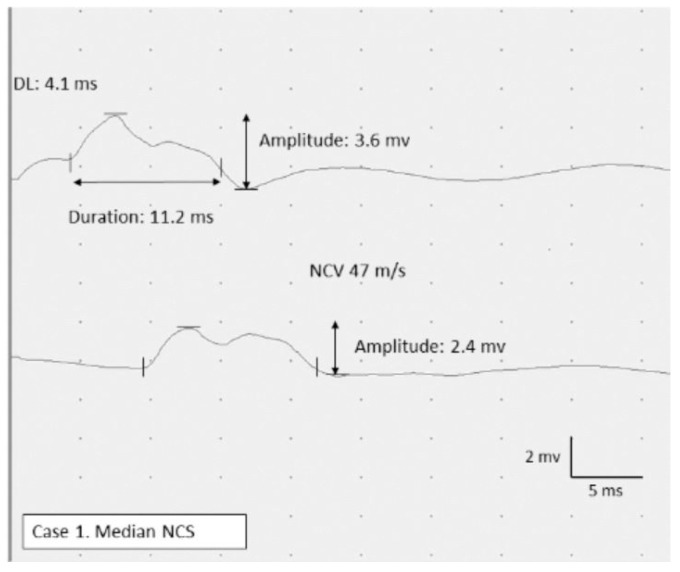
Nerve conduction study. The right median nerve CMAP shows decreased peak-to-peak amplitude (2.8 mv) and prolonged duration (11.2 ms). Only a slight delay in DL and NCV is observed. There is no increased temporal dispersion and conduction block that suggest demyelinating neuropathy. CMAP compound muscle action potential; DL, distal latency; NCV, nerve conduction velocity. Normal values for NCS findings: DL, <3.9 ms; CMAP duration, 7.0 ms; NCV, >53 m/s.

**Figure 3:**
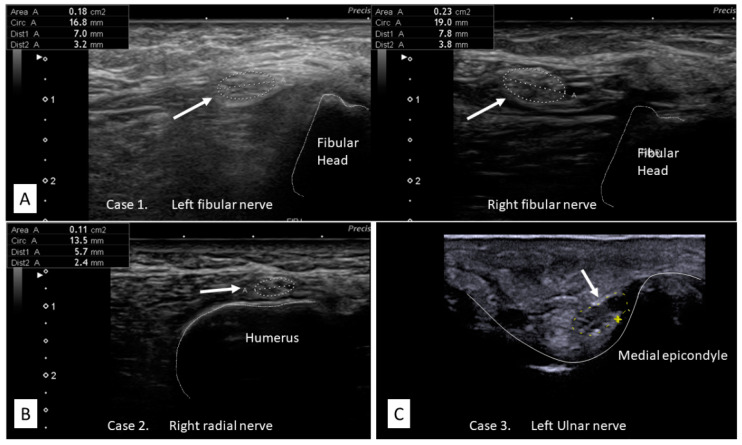
Nerve ultrasound images. (**A**) Case 1: The bilateral fibular nerve shows focal swelling with increased CSA between the fibular head and the popliteal fossa in both legs, suggesting a compression neuropathy at the fibular head (**left**: 18 mm^2^, **right**: 23 mm^2^) *. (**B**) Case 2: The left radial nerve shows focal swelling with increased CSA (11 mm^2^) *, suggesting compression neuropathy at the spiral groove. (**C**) Case 3: The left ulnar nerve shows focal swelling with increased CSA (13 mm^2^) *, suggesting a compression neuropathy at the cubital tunnel. CSA, cross-sectional area. * Normal values for CSA at the evaluated sections of our laboratory: the fibular nerve, 12 mm^2^; the radial nerve, 8 mm^2^; the ulnar nerve, 10 mm^2^.

After being weaned off from mechanical ventilation, the patient was transferred to our rehabilitation unit on day 75. He showed severe generalized muscle weakness (MRC sum score: 27/60) and marked dysesthesia and hypesthesia, predominantly in the distal legs. Furthermore, ankle dorsiflexion was relatively weaker than plantar flexion on both sides (MRC scale: 0/5 for dorsiflexion and 2/5 for plantar flexion, bilaterally), indicative of concomitant bilateral fibular nerve neuropathy, although the findings were not identified during the ICU admission. The patient underwent muscle MRI for the first evaluation of ICUAW. STIR images of the MRI showed diffuse hyperintense lesions predominantly in the truncal and proximal limb-girdle muscles, suggesting denervation or intramuscular edema (Figure 1A). After the examination, neurophysiological studies were performed. NCS revealed sensorimotor polyneuropathy with an absent CMAP on the bilateral fibular and tibial nerves and absent SNAP on the bilateral sural nerve. Furthermore, the right median nerve showed prolonged CMAP duration (11.2 ms), suggesting concomitant CIM (Figure 2). Meanwhile, there were no marked abnormalities in latency and nerve conduction velocity (NCV) that could suggest Guillain–Barré syndrome (GBS) with acute demyelinating polyradiculoneuropathy variant. EMG showed neurogenic changes with denervation potentials in the right deltoid muscle and complete denervation in the bilateral tibialis anterior muscle. NUS confirmed bilateral fibular compression neuropathy showing nerve swelling with an increased cross-sectional area between the fibular head and the popliteal fossa in both legs (Figure 3A). Meanwhile, no abnormalities were found in other nerves, including the brachial plexus on NUS. On the basis of these findings, we diagnosed CINM complicated by bilateral fibular nerve compression neuropathy. He underwent a multidisciplinary rehabilitation program, and three months later, muscle strength and functional ability showed a significant improvement (MRC sum score 41/60, Barthel index: 89/100); however, persistent severe bilateral foot drop was still affecting the patient’s gait performance. A summary of the clinical information is described in Table 1.

### 2.2. Cases 2 and 3

We encountered another two patients with COVID-19-related ICUAW complicated by peripheral nerve injury. Their clinical information is also displayed in Table 1.

During their ICU admission, ICUAW was diagnosed on the basis of generalized muscle weakness and abnormal spontaneous activities in EMG. After being transferred to our unit, the patients underwent further evaluations. Careful physical examinations revealed left wrist drop in case 2 and bilateral first dorsal interosseous muscle atrophy in case 3, suggesting a focal sign of radial nerve neuropathy and ulnar nerve neuropathy. However, the findings were barely detectable due to the generalized muscle weakness and atrophy. In both cases, electrophysiological studies revealed the coexistence of sensorimotor polyneuropathy and myopathic elements. Specifically, both cases showed prolonged CMAP duration in NCS and showed myopathic changes in EMG. On the basis of these findings, we confirmed CINM in both cases. 

Notably, NUS identified compression neuropathy in the left radial nerve in case 2 and bilateral ulnar nerve in case 3. Focal nerve swelling suggesting nerve compression was observed at the left spiral groove in case 2 and at the bilateral cubital tunnel in case 3 (Figure 3B,C). These compression neuropathies were not identified during their ICU admission, as was the case with case 1. Furthermore, NCS did not show typical findings of compression neuropathy due to the underlying sensorimotor polyneuropathy. Regarding MRI, both cases showed massive hyperintense lesions, suggesting intramuscular edema predominantly in the truncal and proximal limb-girdle muscles in STIR imaging. Of note, in case 3, hyperintense lesions were more prominent in T1 imaging than in STIR imaging, suggesting fatty filtration, a typical finding of chronic phase ICUAW (Figure 1B,C).

After the patients received a 3-month multidisciplinary rehabilitation program, an overall functional outcome in both cases achieved a good outcome (Barthel index: 90/100 and 95/100 for cases 2 and 3, respectively); however, in case 2, the severe left wrist drop persisted for more than three months, affecting the patient’s daily activity. 

## 3. Discussion

### 3.1. COVID-19-Related ICUAW

#### 3.1.1. Epidemiology of COVID-19-Related ICUAW

Since early 2020, research on COVID-19-related ICUAW have been rapidly increasing [7,9,21,22]. Several studies have reported that COVID-19 demonstrates various kinds of neurological complications such as headache, stroke, epilepsy, encephalopathy, myositis, and Guillain–Barré syndrome [23,24,25,26]. Nevertheless, ICUAW may be one of the most problematic neuromuscular complications of COVID-19, considering the burden on the patients and healthcare workers originating from its criticalness during the acute phase and poor functional outcome as a long-term after effect [2,14,22]. 

Epidemiological data regarding COVID-19-related ICUAW is still scarce. Recent observational studies on ICU cohorts reported that 4.9–10% of severe COVID-19 cases were diagnosed with CIP, CIM, or CINM confirmed by conventional electrophysiological studies [7,9]. Meanwhile, Van Aerde et al. reported that 72% of the patients were diagnosed with ICUAW using minimum criteria without electrophysiological studies [8]. These results may reflect the fact that non-essential studies were delayed because of the severe nature of the disease, and some patients could die before appropriate electrophysiological evaluation [7]. Hence, at present, COVID-19-related ICUAW might have been underestimated in clinical practice [22].

To date, it is still controversial as to whether COVID-19-related ICUAW is distinct from other types of ICUAW. Several researchers insist on the need for the survey on a larger cohort of COVID-19 cases to solve this issue [7,9,13,14,17,21,22]. Nevertheless, no distinctive features have been reported in COVID-19-related ICUAW regarding basic clinical findings, such as the patient’s background, clinical symptoms, and electrophysiological and pathological studies [7,13,14,17]. In this context, our three cases showed known features of ICUAW. Specifically, the patients had a history of ARDS, septic shock, and MOF, and risk factors such as long-term mechanical ventilation with deep sedation, the use of neuromuscular blocking agents, and corticosteroids therapy. Furthermore, electrophysiological studies showed sensorimotor axonal neuropathy with concomitant myopathic element, which concurred with CINM [1,2,27].

#### 3.1.2. Utility of Electrophysiological Studies in Diagnosing ICUAW 

Several studies have reported that COVID-19-related ICUAW emerges as CIP, CIM, or CINM, as with ordinary ICUAW [7,9,13,14,17,22,28]. CIP is a primary symmetrical sensorimotor axonal polyneuropathy. While the subtype dominantly affects the distal muscles, the proximal muscles, including the respiratory muscles, are usually involved. CIM is a primary myopathy that is not secondary to muscle denervation, and its clinical features are usually similar to those seen in CIP, presenting generalized muscle weakness and difficulty in weaning from the ventilator. CINM is a combined form of CIP and CIM, and the form is possibly more common than CIP and CIM in non-COVID-19 ICUAW [1,2,29].

If the patient is alert and cooperative, the MRC sum score evaluated by a manual muscle test helps diagnose ICUAW on the basis of a score less than 48/60. Additionally, examining sensory disturbance will be a clue to distinguish CIP and CINM from CIM [1,2]. However, it is often challenging to obtain adequate information from physical examinations because of the patient’s conditions such as mechanical ventilation, deep sedation, and the critical illness itself, including coma [1,2]. Furthermore, the defined scale of MRC sum scores above 48/60 could limit the sensitivity to detect subtle changes in muscle function [28,30]. Bax et al. reported that four out of six severe COVID-19 patients were diagnosed with CIP, CIM, or CINM by electrophysiological studies, even though their MRC sum scores were above 48/60 [28]. When these issues are considered, electrophysiological studies are essential to achieve an accurate diagnosis [2,7,14]. 

In our case series, EMG and NCS successfully identified ICUAW, even during ICU admission, and further studies after the discharge diagnosed CINM in all three cases. It is known that NCS shows decreased or absent CMAP amplitudes in all three subtypes, with decreased or absent SNAP amplitudes potentially being a sign of CIP and CINM [1,2,7,14,27]. Furthermore, as shown in our cases, prolonged CMAP duration can be found in CIM and CINM, and particularly, it appears without slowing of nerve conduction, reflecting reduced muscle fiber excitability [22,31]. This specific finding may be useful for excluding other neuromuscular complications with generalized muscle weakness in COVID-19, such as Guillain–Barré syndrome and myositis [25,26]. 

In general, EMG shows varying degrees of fibrillation potentials and positive sharp waves in spontaneous activity assessment in all three subtypes of ICUAW [1,2]. If voluntary activity assessment is available, CIP shows a neurogenic pattern, and CIM shows a myopathic pattern; however, both features could often coexist in one individual [1,2,27]. The specific EMG features of COVID-19-related ICUAW are still uncertain. In fact, we could not figure out specific findings in our cases with conventional EMG evaluation with qualitative assessment. However, according to Martinez et al., quantitative EMG assessment revealed that CIM tended to show more abundant spontaneous activities than other subtypes in COVID-19-related ICUAW [7].

#### 3.1.3. Functional Outcome of COVID-19-Related ICUAW

The evidence regarding the long-term functional outcome in COVID-19-related ICUAW is still under development [7,32]. Regarding non-COVID-19 ICUAW, it is known that it causes long-term effects; although muscle weakness improves in time, functional status and quality of life can be substantially affected over months to years [4,29,33]. Regarding this, Van Aerde et al. reported a similar tendency in COVID-19-related ICUAW on the basis of a short period 3-month observational study focused on hospitalized cases [8]. The study revealed that even though the MRC sum score improved throughout hospitalization, the impact on functional status, measured by ICU mobility score and Barthel index, remained substantial in COVID-19-related ICUAW. Meanwhile, the difference in long-term effects between CIP, CIM, and CINM in COVID-19 cases is still unclear. Past studies on non-COVID-19 ICUAW suggested that CIP tends to result in persistent disability, whereas CIM or CINM possibly achieve complete recovery [2,29]. The relatively good functional outcome of our three cases with CINM might reflect the different prognoses of the subtypes. Recently, Agergaard et al. reported that COVIID-19-related CIM patients could more frequently experience physical fatigue and myalgia as sequelae than other subtypes [21]. When these perspectives are considered, differentiating the ICUAW subtypes may further help predict patient’s long-term outcomes and after-effects in COVID-19 cases.

#### 3.1.4. Utility of Muscle MRI in COVID-19-Related ICUAW

Several studies have reported that muscle MRI provides supportive information about ICUAW through the abnormal signal changes in the muscles [15,34,35]. According to a case series of COVID-19 patients reported by Fernandez et al., STIR images depict muscle denervation edema in CIP and multi-focal intramuscular edema-like signal in CIM as hyperintense lesions. Furthermore, in the chronic phase, T1 images show hyperintense lesions reflecting subsequent fatty infiltration and atrophy of the muscles in both conditions [15,34,36]. In our three cases, the test revealed the abnormalities predominantly in the proximal muscles. Notably, the muscle MRI allowed us to identify the massive muscle damage and reinforce the diagnosis of ICUAW before performing detailed electrophysiological studies in case 1. The chronic signal change in T1 images was obvious in case 3. 

Although MRI is not a primary test for diagnosing ICUAW, it might be an alternative option when the availability of electrophysiological studies is limited. 

### 3.2. Peripheral Nerve Injuries and COVID-19-Related ICUAW

#### 3.2.1. Epidemiology of Peripheral Nerve Injuries in COVID 19 Cases

Recent studies revealed that COVID-19 affects the peripheral nervous system in several ways. Firstly, CIP and CINM cause sensorimotor polyneuropathy, as mentioned above. Furthermore, COVID-19 could trigger immune-mediated neuropathies, such as Guillain–Barré syndrome and Personage–Turner syndrome, and the virus itself might invade the peripheral nerves [15,16,23,25,26,37]. Moreover, peripheral nerve injuries associated with ICU care have been reported as a possible complication in severe COVID-19 cases [15,16,17,38].

In severe COVID-19 pneumonia cases, intensive respiratory care, including long-term deep sedation and immobilization, could increase the chance for unexpected compression on the nerves. In addition, several positioning-related factors, such as exposure to the prone position, could also cause unfavorable pressure or stretching on the nerves and brachial plexus [15,16,17,20]. According to the latest reports, the incidence of peripheral nerve injuries among the COVID-19 patients who underwent ICU care was 14.5–16%, and the involved nerves were the median nerve, ulnar nerve, radial nerve, sciatic nerve, fibular nerve, and brachial plexus [15,16,17,20,38]. Our three cases have a similar background to the reported cases; the patients needed long-term intensive mechanical ventilation with deep sedation and were exposed to the prone position.

#### 3.2.2. ICU Care and Peripheral Nerve Injuries in COVID-19

It is known that the prone position can cause brachial plexus injury due to arm extension accompanied by excessive nerve stretching, although it was not observed in our cases [15,17]. Furthermore, the position could also lead to ulnar nerve injury at the cubital tunnel level, as seen in case 3 [15,19]. Since the ulnar nerve is superficial and relatively unprotected by overlying soft tissue at the level, inappropriate placement of the elbow can produce unfavorable external compression on the nerve and lead to nerve injury [39]. In terms of cases 1 and 2, fibular nerve and radial nerve injuries are less associated with the prone position [15,19]. Meanwhile, several conditions relevant to ICU care can be the risks for these nerve injuries. Suboptimal knee positioning can enhance the compression of the fibular nerve at the fibular head level [15,19]. Continuous use of an automatically cycled blood pressure cuff can cause radial nerve injury [19,40]. We speculated that unexpected compressions or stretching associated with positioning or medical procedures might have resulted in the peripheral nerve injuries in our cases.

In addition to intensive respiratory care and positionings, other procedures can also cause peripheral nerve injuries. ECMO, for example, is reported as a possible cause of lower limb peripheral nerve injuries, although no COVID-19 cases have been reported [40,41,42,43]. According to the literature, bulky cannulas placed on the femoral artery and vein can damage the femoral nerve [40,41]. Furthermore, compartment syndrome, a possible complication of the procedure, can also cause fibular, tibial, and sural nerve injuries on the same side of cannulation [42,43]. Fortunately, case 1 did not show the peripheral nerve injuries associated with ECMO, although the patient underwent the procedure. Nevertheless, given the increasing usage of ECMO in COVID-19 cases, the peripheral nerve complications should be kept in mind.

#### 3.2.3. Diagnostic Challenges of Peripheral Nerve Injuries and Utility of NUS

The diagnosis of peripheral nerve injuries can be greatly affected by coexisting ICUAW. Firstly, the massive distribution of muscle weakness and sensory disturbance substantially masks typical neurological signs of peripheral nerve injuries [15]. In our cases, careful physical examinations performed by experienced neurologists barely detected focal neurological deficits: bilateral foot drop in case 1, left wrist drop in case 2, and bilateral first dorsal interosseous muscle atrophy in case 3. Furthermore, typical NCS findings of compression neuropathies can also be masked by the axonal loss due to superimposed sensorimotor polyneuropathy, as seen in our cases.

Notably, NUS successfully confirmed the compression neuropathies through morphological changes in the nerves. In general, compression neuropathies demonstrate swelling of the nerve and fascicles, loss of fascicular structure, and alterations of echogenicity of the nerves [44,45]. Our cases showed focal nerve swelling with increased nerve cross-sectional area, and the findings helped diagnose and localize the affected lesions. On the basis of our experience, morphological evaluations through NUS will be a useful option to detect concomitant peripheral nerve injuries in COVID-19 cases even under the influence of ICUAW. On another note, a careful neurological examination is also essential to trigger further evaluations with NUS and proceed to early interventions. Physicians should be vigilant for even a subtle sign of focal neurological deficits, such as laterality and disproportionate weakness or sensory disturbance in a certain nerve area. 

#### 3.2.4. Functional Outcome of Peripheral Nerve Injuries and Appropriate Interventions

In the present cases, we introduced a multidisciplinary rehabilitation program after the diagnosis of peripheral nerve injuries. However, the bilateral foot drop in case 1 and the left wrist drop in case 2 persisted for more than three months, affecting the patient’s functional ability, even though the muscle weakness due to ICUAW showed significant improvement. Several researchers have insisted that peripheral nerve injury could affect the quality of life in COVID-19 survivors, as seen in our cases [15,16,17,38]. Hence, besides rehabilitative interventions, proper preventions for this complication, such as frequent mobilization and appropriate positionings, are essential to improve the patient’s functional outcome [16,19]. In this regard, peripheral nerve injuries relevant to sedation and positionings have been well documented as intraoperative complications during general anesthetic surgery [19,46]. The preventive measures used in this field might also be useful in managing severe COVID-19 cases.

## 4. Conclusions

During the acute phase of severe COVID-19 infection, most medical attention is generally assigned to critical care management, and neuromuscular complications such as ICUAW and peripheral nerve injuries could be underestimated [6,7,11]. When starting post-ICU care for COVID-19 cases, the combination of electrophysiological and imaging studies will be beneficial for evaluating the neuromuscular condition of the patients and help start appropriate interventions.

## Figures and Tables

**Table 1:** Clinical information of three cases.

	Case 1	Case 2	Case 3
Age	52	77	52
Sex	Male	Male	Male
ICU stay (days)	82	70	44
Mechanical ventilation (days)	60	50	39
Comorbidities	ARDS, septic shock, MOF	ARDS, septic shock, MOF	ARDS
NBA	+	+	+
Corticosteroids	+	+	+
Additional treatment	ECMO	CHDF	None
Type of ICUAW	CINM	CINM	CINM
Peripheral nerve injury	bilateral fibular nerve	left radial nerve	bilateral ulnar nerve
(site)	(fibular head)	(spiral groove)	(cubital tunnel)
Focal neurological deficits	bilateral foot drop	left wrist drop	bilateral FDI atrophy
Nerve conduction study	sensorimotor poly neuropathy	sensorimotor poly neuropathy	sensorimotor poly neuropathy
Prolonged CMAP duration	+	+	+
Electromyography	neurogenic changes with Fib/PSW	myopathic changes with Fib/PSW	myopathic changes with Fib/PSW
Nerve ultrasound	focal enlargement in the bilateral fibular nerve	focal enlargement in the left radial nerve	focal enlargement in the left ulnar nerve
Muscle MRI (STIR image)	diffuse hyperintense lesions	diffuse hyperintense lesions	diffuse hyperintense lesions
MRC sum-score			
On admission	34	27	38
Three months later	51	41	54
Barthel index			
On admission	5	5	11
Three months later	95	90	95

ARDS, acute respiratory distress syndrome; CHDF, continuous hemodiafiltration; CINM, critical illness neuromyopathy; CMAP, compound muscle action potential; ECMO, extracorporeal membrane oxygenation; FDI, first dorsal interosseous muscle; Fib/PSW, fibrillations/positive sharp wave; ICUAW, intensive care unit-acquired weakness; NBA, neuromuscular blocking agent; SNAP, sensory nerve action potential; STIR, short T1 inversion recovery.

## References

[1] Stevens R.D., Marshall S.A., Cornblath D.R., Hoke A., Needham D.M., De Jonghe B., Ali N.A., Sharshar T. (2009). A framework for diagnosing and classifying intensive care unit-acquired weakness. Crit. Care Med..

[2] Latronico N., Bolton C.F. (2011). Critical illness polyneuropathy and myopathy: A major cause of muscle weakness and paralysis. Lancet Neurol..

[3] Zhou C., Wu L., Ni F., Ji W., Wu J., Zhang H. (2014). Critical illness polyneuropathy and myopathy: A systematic review. Neural Regen. Res..

[4] Sidiras G., Patsaki I., Karatzanos E., Dakoutrou M., Kouvarakos A., Mitsiou G., Routsi C., Stranjalis G., Nanas S., Gerovasili V. (2019). Long term follow-up of quality of life and functional ability in patients with ICU acquired Weakness—A post hoc analysis. J. Crit. Care.

[5] Grasselli G., Zangrillo A., Zanella A., Antonelli M., Cabrini L., Castelli A., Cereda D., Coluccello A., Foti G., Fumagalli R. (2020). Baseline Characteristics and Outcomes of 1591 Patients Infected with SARS-CoV-2 Admitted to ICUs of the Lombardy Region, Italy. J. Am. Med. Assoc..

[6] Stam H.J., Stucki G., Bickenbach J. (2020). Covid-19 and post intensive care syndrome: A call for action. J. Rehabil. Med..

[7] Cabañes-martínez L., Villadóniga M., González-rodríguez L., Araque L., Díaz-cid A., Ruz-caracuel I., Pian H., Sánchez-alonso S., Fanjul S., Regidor I. (2020). Neuromuscular involvement in COVID-19 critically ill patients. Clin. Neurophysiol..

[8] Van Aerde N., Van den Berghe G., Wilmer A., Gosselink R., Hermans G., Meersseman P., Gunst J., Aerts V., Balthazar T., Barbé A. (2020). Intensive care unit acquired muscle weakness in COVID-19 patients. Intensive Care Med..

[9] Frithiof R., Rostami E., Kumlien E., Virhammar J., Fällmar D., Hultström M., Lipcsey M., Ashton N., Blennow K., Zetterberg H. (2021). Critical illness polyneuropathy, myopathy and neuronal biomarkers in COVID-19 patients: A prospective study. Clin. Neurophysiol..

[10] Alhazzani W., Møller M.H., Arabi Y.M., Loeb M., Gong M.N., Fan E., Oczkowski S., Levy M.M., Derde L., Dzierba A. (2020). Surviving Sepsis Campaign: Guidelines on the Management of Critically Ill Adults with Coronavirus Disease 2019 (COVID-19).

[11] McClafferty B., Umer I., Fye G., Kepko D., Kalayanamitra R., Shahid Z., Ramgobin D., Cai A., Groff A., Bhandari A. (2020). Approach to critical illness myopathy and polyneuropathy in the older SARS-CoV-2 patients. J. Clin. Neurosci..

[12] Mehta P., McAuley D.F., Brown M., Sanchez E., Tattersall R.S., Manson J.J. (2020). COVID-19: Consider cytokine storm syndromes and immunosuppression. Lancet.

[13] Bagnato S., Boccagni C., Marino G., Prestandrea C., Agostino T.D., Rubino F. (2020). Critical illness myopathy after COVID-19. Int. J. Infect. Dis..

[14] Tankisi H., Tankisi A., Harbo T., Markvardsen L.K., Andersen H., Pedersen T.H. (2020). Critical illness myopathy as a consequence of Covid-19 infection. Clin. Neurophysiol..

[15] Fernandez C.E., Franz C.K., Ko J.H., Walter J.M., Koralnik I.J., Ahlawat S., Deshmukh S. (2021). Imaging Review of Peripheral Nerve Injuries in Patients with COVID-19. Radiology.

[16] Malik G.R., Wolfe A.R., Soriano R., Rydberg L., Wolfe L.F., Deshmukh S., Ko J.H., Nussbaum R.P., Dreyer S.D., Jayabalan P. (2020). Injury-prone: Peripheral nerve injuries associated with prone positioning for COVID-19-related acute respiratory distress syndrome. Br. J. Anaesth..

[17] Nasuelli N.A., Pettinaroli R., Godi L., Savoini C., De Marchi F., Mazzini L., Crimaldi F., Pagni A., Pompa C.P., Colombo D. (2020). Critical illness neuro-myopathy (CINM) and focal amyotrophy in intensive care unit (ICU) patients with SARS-CoV-2: A case series. Neurol. Sci..

[18] Rubinos C., Ruland S. (2016). Neurologic Complications in the Intensive Care Unit. Curr. Neurol. Neurosci. Rep..

[19] Winfree C.J., Kline D.G. (2005). Intraoperative positioning nerve injuries. Surg. Neurol..

[20] Le M.Q., Rosales R., Shapiro L.T., Huang L.Y. (2020). The down Side of Prone Positioning: The Case of a Coronavirus 2019 Survivor. Am. J. Phys. Med. Rehabil..

[21] Agergaard J., Leth S., Pedersen T.H., Harbo T., Blicher J.U., Karlsson P., Østergaard L., Andersen H., Tankisi H. (2021). Myopathic changes in patients with long-term fatigue after COVID-19. Clin. Neurophysiol..

[22] Tankisi H. (2021). Critical illness myopathy and polyneuropathy in Covid-19: Is it a distinct entity?. Clin. Neurophysiol..

[23] Berlit P., Bösel J., Gahn G., Isenmann S., Meuth S.G., Nolte C.H., Pawlitzki M., Rosenow F., Schoser B., Thomalla G. (2020). “Neurological manifestations of COVID-19”—Guideline of the German society of neurology. Neurol. Res. Pract..

[24] Pinzon R.T., Wijaya V.O., Buana R.B., Al Jody A., Nunsio P.N. (2020). Neurologic characteristics in coronavirus disease 2019 (COVID-19): A systematic review and meta-analysis. Front. Neurol..

[25] Tatu L., Nono S., Grácio S., Koçer S. (2020). Guillain–Barré syndrome in the COVID-19 era: Another occasional cluster?. J. Neurol..

[26] Harapan B.N., Yoo H.J. (2021). Neurological symptoms, manifestations, and complications associated with severe acute respiratory syndrome coronavirus 2 (SARS-CoV-2) and coronavirus disease 19 (COVID-19). J. Neurol..

[27] Bolton C.F. (2005). Neuromuscular manifestations of critical illness. Muscle Nerve.

[28] Bax F., Lettieri C., Marini A., Pellitteri G., Surcinelli A., Valente M., Budai R., Patruno V., Gigli G.L. (2021). Clinical and neurophysiological characterization of muscular weakness in severe COVID-19. Neurol. Sci..

[29] Guarneri B., Bertolini G., Latronico N. (2008). Long-term outcome in patients with critical illness myopathy or neuropathy: The Italian multicentre CRIMYNE study. J. Neurol. Neurosurg. Psychiatry.

[30] Vanhorebeek I., Latronico N., Van den Berghe G. (2020). ICU-acquired weakness. Intensive Care Med..

[31] Goodman B.P., Harper C.M., Boon A.J. (2009). Prolonged compound muscle action potential duration in critical illness myopathy. Muscle Nerve.

[32] Curci C., Negrini F., Ferrillo M., Bergonzi R., Bonacci E., Camozzi D.M., Ceravolo C., De Franceschi S., Guarnieri R., Moro P. (2021). Functional outcome after inpatient rehabilitation in post-intensive care unit COVID-19 patients: Findings and clinical implications from a real-practice retrospective study. Eur. J. Phys. Rehabil. Med..

[33] Fan E., Cheek F., Chlan L., Gosselink R., Hart N., Herridge M.S., Hopkins R.O., Hough C.L., Kress J.P., Latronico N. (2014). An official American Thoracic Society Clinical Practice guideline: The diagnosis of intensive care unit-acquired weakness in adults. Am. J. Respir. Crit. Care Med..

[34] Dinh A., Carlier R., Descatha A. (2016). Critical illness myopathy and whole body mri. Intensive Care Med..

[35] Ten Dam L., van der Kooi A.J., Verhamme C., Wattjes M.P., de Visser M. (2016). Muscle imaging in inherited and acquired muscle diseases. Eur. J. Neurol..

[36] Kayim Yildiz O., Yildiz B., Avci O., Hasbek M., Kanat S. (2021). Clinical, Neurophysiological and Neuroimaging Findings of Critical Illness Myopathy after COVID-19. Cureus.

[37] Coraci D., Fusco A., Frizziero A., Giovannini S., Biscotti L., Padua L. (2020). Global approaches for global challenges: The possible support of rehabilitation in the management of COVID-19. J. Med. Virol..

[38] Needham E., Newcombe V., Michell A., Thornton R., Grainger A., Anwar F., Warburton E., Menon D., Trivedi M., Sawcer S. (2020). Mononeuritis multiplex: An unexpectedly frequent feature of severe COVID-19. J. Neurol..

[39] Bickler P.E., Schapera A., Bainton C.R. (1991). Acute Radial Nerve Injury from Use of an Automatic Blood Pressure Monitor. Obstet. Anesth. Dig..

[40] Jang A.Y., Oh Y.J., Lee S.I., Lim O.K., Suh S.Y. (2020). Femoral neuropathy following venoarterial-extracorporeal membrane oxygenation therapy: A case report. BMC Cardiovasc. Disord..

[41] Rieg S., von Cube M., Kalbhenn J., Utzolino S., Pernice K., Bechet L., Baur J., Lang C.N., Wagner D., Wolkewitz M. (2020). COVID-19 in-hospital mortality and mode of death in a dynamic and non-restricted tertiary care model in Germany. PLoS ONE.

[42] Go J.Y., Min Y.S., Jung T. (2014). Du Delayed onset of acute limb compartment syndrome with neuropathy after venoarterial extracorporeal membrane oxygenation therapy. Ann. Rehabil. Med..

[43] Aydin Ş., Pazarci N., Akan O., Büyükkale S., Bakan N.D., Sayar A. (2019). A case report of a drop foot developed after common femoral artery cannulation for venoarterial extracorporeal membrane Oxygenation. Arch. Neuropsychiatry.

[44] Hobson-Webb L.D., Padua L. (2016). Ultrasound of Focal Neuropathies. J. Clin. Neurophysiol..

[45] Padua L., Coraci D., Erra C., Pazzaglia C., Paolasso I., Loreti C., Caliandro P., Hobson-Webb L.D. (2016). Carpal tunnel syndrome: Clinical features, diagnosis, and management. Lancet Neurol..

[46] Kwee M.M., Ho Y.H., Rozen W.M. (2015). The prone position during surgery and its complications: A systematic review and evidence-based guidelines. Int. Surg..

